# Cardiac Sarcoidosis: Pathophysiology and Diagnostic–Therapeutic Processes

**DOI:** 10.31083/RCM43535

**Published:** 2026-01-19

**Authors:** Serena Antonaci, Zumrud Ismibayli, Silvana De Martino, Giulia Azzurra De Santis, Kareem Salame, Marco Russo

**Affiliations:** ^1^Cardiology Unit, Sacro Cuore di Gesù Hospital, 73014 Gallipoli, Italy; ^2^Cardiology Unit, XMSK Hospital, AZ 1004 Baku, Azerbaijan; ^3^Medicine Department, The Sheba Medical Center at Tel Hashomer, 5265601 Tel Aviv, Israel

**Keywords:** cardiac sarcoidosis, inflammatory heart disease, heart failure, heart block, ventricular arrhythmias

## Abstract

Sarcoidosis is a rare inflammatory disorder of unknown etiology, characterized by the formation of non-caseating granulomas in affected organs. Additionally, sarcoidosis typically involves multiple systems, with the lungs and thoracic lymph nodes being most commonly affected. While many cases are self-limited and resolve spontaneously, cardiac involvement, although relatively uncommon, can be particularly severe. Indeed, cardiac sarcoidosis may lead to life-threatening arrhythmias, severe heart failure, or sudden cardiac death, significantly impacting prognosis. Meanwhile, the heterogeneity of presentation and disease course can make diagnosis and treatment challenging. An endomyocardial biopsy (EMB) is considered the gold standard for diagnosing cardiac sarcoidosis (CS); despite its high specificity, the sensitivity of this technique is low owing to the often focal and patchy cardiac involvement in sarcoidosis. New imaging techniques, such as fluorine-18 fluorodeoxyglucose positron emission tomography (FDG-PET) and cardiac magnetic resonance (CMR) imaging, can provide valuable information for the accurate diagnosis of CS and can be useful for evaluating treatment response and prognosis. Immunosuppressive treatments, particularly corticosteroids, are considered the cornerstone of therapy for CS. However, randomized clinical trials are lacking, and treatment decisions are based on cohort studies and consensus opinions. Moreover, the optimal strategy for determining when to initiate, how long to continue, and what dosage to use for immunosuppressive therapy remains uncertain.

## 1. Introduction

Cardiac sarcoidosis (CS) is an infiltrative heart disease of unknown etiology, characterized by granulomatous inflammation of the myocardial tissue. The progression and severity of the disease can vary widely.

Clinical manifestations can include impaired atrioventricular conduction, ventricular arrhythmias, congestive heart failure, and sudden cardiac death. Although the disease can have a significant clinical impact, diagnosis is often challenging since the various diagnostic techniques have limited sensitivity and specificity, particularly for isolated cardiac forms.

The purpose of this review is to assess the present knowledge about the pathophysiology and diagnostic–therapeutic pathways of CS.

## 2. Epidemiology

The prevalence and incidence of sarcoidosis show considerable variation across regions, sexes, and racial groups. Globally, prevalence rates range from 1 to 160 cases per 100,000 individuals, while the annual incidence is estimated between 0.5 and 11.5 cases per 100,000. Prevalence is highest in Scandinavian countries, especially Sweden, while countries in East Asia, such as South Korea, report much lower incidence rates. These disparities reflect a complex interplay of genetic, environmental, and healthcare access factors. In the United States, non-Hispanic Black individuals, particularly women, experience the highest rates of systemic sarcoidosis and also demonstrate increased mortality compared to other racial groups. Women, in general, are more frequently diagnosed with sarcoidosis, which may be partly attributable to later age at diagnosis. Some evidence suggests menopause-related factors, such as decreased lung function, could contribute, although the role of sex hormones in disease onset and progression remains an area requiring further study [1, 2].

CS occurs in approximately 3% to 10% of patients with systemic sarcoidosis, although autopsy studies suggest that up to 25% may have subclinical cardiac involvement. The recognition of CS has increased in recent years, driven by advancements in imaging, updated diagnostic criteria, and greater clinical awareness. However, estimating the true prevalence remains difficult due to its heterogeneous manifestations and the presence of isolated or subclinical disease [1].

Geographic variation in CS is also notable. In Finland, the annual detection of CS increased 20-fold between 1988 and 2021. In Japan, CS is considered particularly prevalent, and cardiac lesions were found to be the main cause of sarcoidosis-related death at autopsy [3, 4]. Kandolin *et al*. [5] reported a detection rate of 0.31 per 100,000 adults in Finland between 2008 and 2012, with a CS prevalence of 2.2 per 100,000 in 2012. Remarkably, 65% of patients in this cohort had isolated cardiac involvement, while the remaining 35% had extracardiac disease [5].

Prevalence estimates for isolated CS vary widely, between 3.2% and 54%, depending on diagnostic criteria and study populations. These patients often exhibit more severe arrhythmias and worse left ventricular systolic function compared to those with systemic disease [1, 6].

Terasaki *et al*. [7] noted that the onset of CS typically occurs between the ages of 25 and 45, with an additional peak around age 50 in Japan and Scandinavian countries, supporting a biphasic age distribution.

Demographic factors—including age, sex, and ethnicity—play a critical role in the diagnosis, progression, and outcomes of both systemic sarcoidosis and CS. While women are more frequently diagnosed with systemic disease, men are more likely to present with cardiac involvement at diagnosis and may experience more severe forms [8].

These disparities highlight the need for greater awareness, earlier screening in high-risk populations, and equitable access to specialized care and diagnostics. The continued evolution of diagnostic imaging and clinical criteria will be key to improving detection and outcomes across diverse demographic groups.

## 3. Pathogenesis

The pathogenesis of sarcoidosis, including its cardiac manifestations, is believed to result from an interplay of genetic susceptibility, environmental exposures, and a dysregulated immune response. Genetic studies have identified several *HLA* alleles that play a significant role in sarcoidosis susceptibility. Notably, HLA-DRB1*0101, DQA1*0101, and DQB1*0501 are strongly associated with increased risk of the disease, particularly in individuals of European descent. These alleles are involved in antigen presentation and immune response regulation, which are key factors in the pathogenesis of sarcoidosis. Recent genome-wide association studies have further elucidated the polygenic nature of sarcoidosis, highlighting the involvement of various immune-related genes in disease development [9].

Environmental and infectious triggers are implicated as initiating antigens in genetically predisposed individuals. Proposed infectious agents include *Propionibacterium acnes* (frequently isolated from sarcoid lesions), *Mycobacterium tuberculosis*, *Borrelia burgdorferi*, *Corynebacterium spp*., and various viruses (*Epstein-Barr virus*, *Cytomegalovirus*, *Coxsackie B*). However, no microbial pathogen has been consistently isolated or cultured, and direct causation has not been definitively proven [10, 11]. Non-infectious occupational exposures have been proposed as potential contributors to sarcoidosis. The ACCESS study identified associations with certain environments, such as agricultural and manufacturing settings, while subsequent studies have further explored exposures to dusts, metals, and industrial particulates in various high-risk occupations [12, 13]. Interestingly, smoking, though typically considered a risk factor for many inflammatory diseases, has been found to reduce the risk of developing sarcoidosis in some studies. The precise mechanisms are not well understood, but may involve immune modulation. However, once sarcoidosis is established, smoking is detrimental, as it can worsen pulmonary involvement and overall disease progression, particularly in the lung and heart [14].

The key pathological feature of sarcoidosis is the formation of non-caseating granulomas, which consist of aggregates of macrophages, epithelioid cells, and T lymphocytes. The immunologic cascade begins when antigen-presenting cells (e.g., macrophages, dendritic cells) present antigens to naïve CD4+ T cells, promoting differentiation primarily into Th1 and Th17 cells. These T cells secrete proinflammatory cytokines such as interleukin-2 (IL-2), interferon-gamma (IFN-γ), tumor necrosis factor-alpha (TNF-α), and IL-12, which recruit and activate macrophages and lymphocytes to form granulomas. Macrophages differentiate into epithelioid cells and may fuse into multinucleated giant cells, forming the core of granulomatous lesions. Over time, a shift from Th1 to Th2 dominance may occur in chronic sarcoidosis, with the secretion of IL-4, IL-10, and transforming growth factor (TGF)-β, leading to fibroblast activation and the subsequent deposition of extracellular matrix, which contributes to tissue fibrosis. IL-6, found in high concentrations in early disease, sustains inflammation through T cell proliferation [1, 15].

Recent studies indicate that granuloma formation may also be influenced by intrinsic changes in macrophages themselves. When the immune system is unable to clear an antigen or stimulus, persistent activation of the mechanistic target of rapamycin complex 1 (mTORC1) in macrophages promotes their proliferation, metabolic reprogramming, and resistance to apoptosis. This results in macrophages transforming into hypertrophic, epithelioid cells that contribute to granuloma formation. mTORC1 activation enhances glycolysis and oxidative phosphorylation, providing the energy necessary for this process. This mechanism underscores a cell-intrinsic pathway for granuloma development, which complements the traditional immune-driven model, and may be relevant to various granulomatous diseases, including sarcoidosis [16].

In CS, granulomas typically form in a patchy, multifocal pattern across all heart layers, with a preference for the left ventricular myocardium, particularly the basal interventricular septum. Histologically, the disease progresses through three overlapping phases beginning with granulomatous inflammation, which is associated with tissue edema, detectable on cardiac magnetic resonance (CMR), and early myocardial dysfunction. As the inflammation subsides, it is replaced by fibrous tissue, leading to fibrosis and subsequent scar formation. Inflammatory infiltrates can disrupt conduction pathways, leading to conduction abnormalities, and create a substrate for ventricular tachycardia (VT). Cytokine-driven damage and oxidative stress further impair cardiomyocyte function, contributing to progressive myocardial dysfunction [1].

## 4. Clinical Presentation

Sarcoidosis is a systemic condition that can affect almost any organ (lymph nodes, lungs, eyes, skin, joints, liver, spleen, parathyroid glands, kidneys, nervous system, heart) [17]. However, lungs and mediastinal lymph nodes involvement is by far the most prevalent, occurring in over 90% of cases [18]. Consequently, respiratory symptoms, such as cough (productive or non-productive) and dyspnea with dry crackles on physical examination, typically constitute the initial clinical presentation.

Two clinical syndromes are considered highly specific for sarcoidosis, and their recognition can significantly aid in establishing the diagnosis. Löfgren’s syndrome is characterized by the acute onset of erythema nodosum, bilateral ankle arthritis, and hilar lymphadenopathy on chest radiography [19]. Heerfordt-Waldenström syndrome, a less common manifestation, presents with acute bilateral parotid gland enlargement, anterior uveitis, and facial nerve palsy [20].

Cutaneous manifestations offer significant insight into systemic sarcoidosis involvement. Specific sarcoid skin lesions, resulting from non-caseating granulomas, include papules (predominantly located on the head, around the eyes, neck, and nasolabial folds), plaques (commonly found on the shoulders and back), and subcutaneous nodules (distributed on the trunk and extremities), lupus pernio (infiltrative red to violaceous plaques affecting ears, nose, cheeks and fingers, simulating frostbite). Conversely, erythema nodosum, the most common cutaneous manifestation in sarcoidosis, is considered a nonspecific lesion because it may also be seen in a variety of other systemic conditions (septal panniculitis without granuloma is its cardinal histopathology) [21].

Symptomatic cardiac involvement is reported in approximately 2–5% of patients with sarcoidosis, even though autoptic [22] and cardiac magnetic resonance [23, 24] studies indicate myocardial involvement in 25% to 30% of all sarcoid patients and even in 9% of patients without symptoms or electrocardiogram (ECG) abnormalities. The prevalence of isolated CS is approximately 20% to 25% [25], which correlates with more serious disease than cardiac involvement associated with concomitant extracardiac disease [26].

Clinical manifestations depend on location, extent, and disease activity [27].

Palpitations, pre-syncope, and syncope are frequent symptoms at presentation because conduction abnormalities and ventricular arrhythmias (VA) are the most common manifestations in CS. Also, sudden cardiac death can be the first manifestation of the disease [28].

Dyspnoea and other signs and symptoms of heart failure reflect an extensive myocardial involvement. Systolic dysfunction and diastolic impairment with increased filling pressures due to edematous or fibrotic left ventricular walls both contribute to heart failure (HF) onset (with both reduced or preserved ejection fraction). HF represents the most important cause of death in patients with CS, accounting for 25% of mortality in some studies [29].

Severe cardiac valve involvement is uncommon (<3%) [22, 30]. Mitral regurgitation may result from left ventricular or mitral annular dilatation, or from sarcoid infiltration of the papillary muscles or valve leaflets [31, 32], mandating mitral valve replacement in some cases [33]. Aortic, tricuspid, or pulmonary valve involvement is less common [30].

Right ventricle (RV) involvement is variable, ranging from 6% to 65% in studies, and is generally associated with poor outcome [34].

Direct granulomatous infiltration of RV walls and pre- or post-capillary pulmonary hypertension (due respectively to lung fibrosis and elevated left ventricular filling pressures due to systolic and/or diastolic impairment) can both contribute to RV dysfunction.

Chest pain resembling angina may occur and is most commonly attributed to a reduction in coronary flow reserve secondary to microvascular compression [35]. However, granulomatous coronary arteritis should also be considered as a potential underlying mechanism [36]. In rare cases, CS may present with clinical features consistent with acute myocardial infarction, with coronary angiography revealing either normal findings, dissection, or complete occlusion of a single coronary artery, coronary arterial aneurysms, or coronary spasm [32, 37, 38, 39].

Pericardial infiltration can result in the development of pericardial effusion and, in rare instances, constrictive pericarditis [40]. In the majority of cases, pericardial involvement is associated with myocardial sarcoid infiltration [41].

## 5. Diagnosis of CS

### 5.1 Laboratory Tests

No specific biomarkers are known for the diagnosis, monitoring, or quantification of CS. Most biomarkers are elevated in patients with sarcoidosis [42]—serum angiotensin converting enzyme, serum soluble interleukin-2-receptor, lysozyme, neopterin, serum amyloid A, troponins, and brain natriuretic peptide (BNP). It could provide evidence suggesting CS, but they lack sensitivity and specificity [43]. Angiotensin-converting enzyme levels have traditionally been used in the diagnosis of sarcoidosis, but their relevance in the context of CS remains uncertain: serum angiotensin-converting enzyme is raised in approximately 60% of patients with systemic sarcoidosis, but only in a minority of those with isolated CS.

Circulating cardiac troponin T, if elevated at diagnosis, responds rapidly to prednisone and may have a prognostic value [44].

C-reactive protein is elevated in patients with sarcoidosis and VT or sarcoidosis with HF compared with patients without active CS [45].

Circulating micro-RNAs play an important role as diagnostic and prognostic biomarkers in cancer and cardiovascular disease. These microRNAs have a regulatory role in the immune system: altered levels of microRNA-126 and microRNA-223 cause the overstimulation of T cells. Real-time polymerase chain reaction showed that microRNA-126 and microRNA-223 in peripheral blood is considerably increased in patients with CS compared with the blood of healthy controls, and they could be potential diagnostic biomarkers for CS and potential therapeutic targets in CS [46].

### 5.2 Electrocardiogram

A standard 12-lead ECG is a useful tool for screening cardiac involvement in patients with sarcoidosis. The Heart Rhythm Society (HRS) consensus statement recommends that individuals with biopsy-confirmed extracardiac sarcoidosis should undergo electrocardiographic evaluation to assess for cardiac involvement (Class I recommendation) [47]. Electrocardiographic abnormalities have been reported in approximately 20% to 31% of patients with sarcoidosis [48, 49].

Atrioventricular conduction disorders are the most common manifestation [50], ranging from first to third degree atrioventricular block (AVB), resulting from infiltration of the basal septum by inflammatory granulomas or fibrosis, or involvement of the nodal artery [30].

Unexplained high-degree AVB in young individuals should prompt evaluation for CS: a prospective analysis of 32 patients aged ≤60 years with unexplained AVB revealed that 34% had undiagnosed CS [50].

Other conduction system abnormalities include nonspecific interventricular conduction delay and complete bundle branch block: at diagnosis, 26–43% of individuals have right bundle branch block on ECG [5].

VAs are the second most common manifestation [5], including multifocal or frequent premature ventricular contractions, VT, and ventricular fibrillation. The underlying mechanism of VAs in CS depends on the inflammatory to fibrotic phase of granulomatous infiltration: most of them are due to reentrant mechanisms around areas of scar; others are secondary to non-reentrant mechanisms (i.e., abnormal automaticity and triggered activity) related to myocardial inflammation. In patients with CS, ablation studies show a complex arrhythmogenic substrate involving the Purkinje system and intramural or epicardial location in both ventricles, even without active inflammation [51].

Atrial arrhythmias—including atrial fibrillation (the most frequently observed), atrial flutter, and atrial tachycardia—may also occur in patients with CS. These arrhythmias can result from atrial dilatation secondary to elevated left ventricular end-diastolic pressure in the setting of ventricular dysfunction, or from direct atrial involvement by granulomatous inflammation or fibrotic scar tissue. In a retrospective cohort study of 100 patients with CS, atrial arrhythmias were documented in 32% of individuals over a mean follow-up period of 5.8 years [52].

Other ECG findings associated with cardiac involvement include: fragmented QRS complex, prolonged QRS, abnormal signal-averaged ECG, epsilon waves, T wave alternans, higher T wave amplitude in AVR, T-wave inversion, axis deviation and abnormal Q waves [53, 54, 55, 56, 57, 58].

Resting electrocardiography demonstrates limited sensitivity for detecting cardiac involvement; therefore, a normal ECG does not exclude the presence of CS. In a study of 112 patients with biopsy-confirmed extra-CS, 33 patients (29%) exhibited normal ECG and/or echocardiographic results (normal group), whereas 79 patients (71%) displayed abnormalities on ECG and/or transthoracic echocardiography (abnormal group). Among these, 6 of the 33 patients (18%) in the normal group and 43 of the 79 patients (59%) in the abnormal group were diagnosed with CS according to the Japanese guidelines [59].

### 5.3 Thoracic Imaging

Chest radiographs (chest X-ray - CXR) are found to be abnormal in nearly 90% of patients with pulmonary sarcoidosis at presentation [60]. Lymphadenopathy is the most frequently observed radiographic finding, present in more than two-thirds of patients [61, 62, 63].

**Fig. 1.**
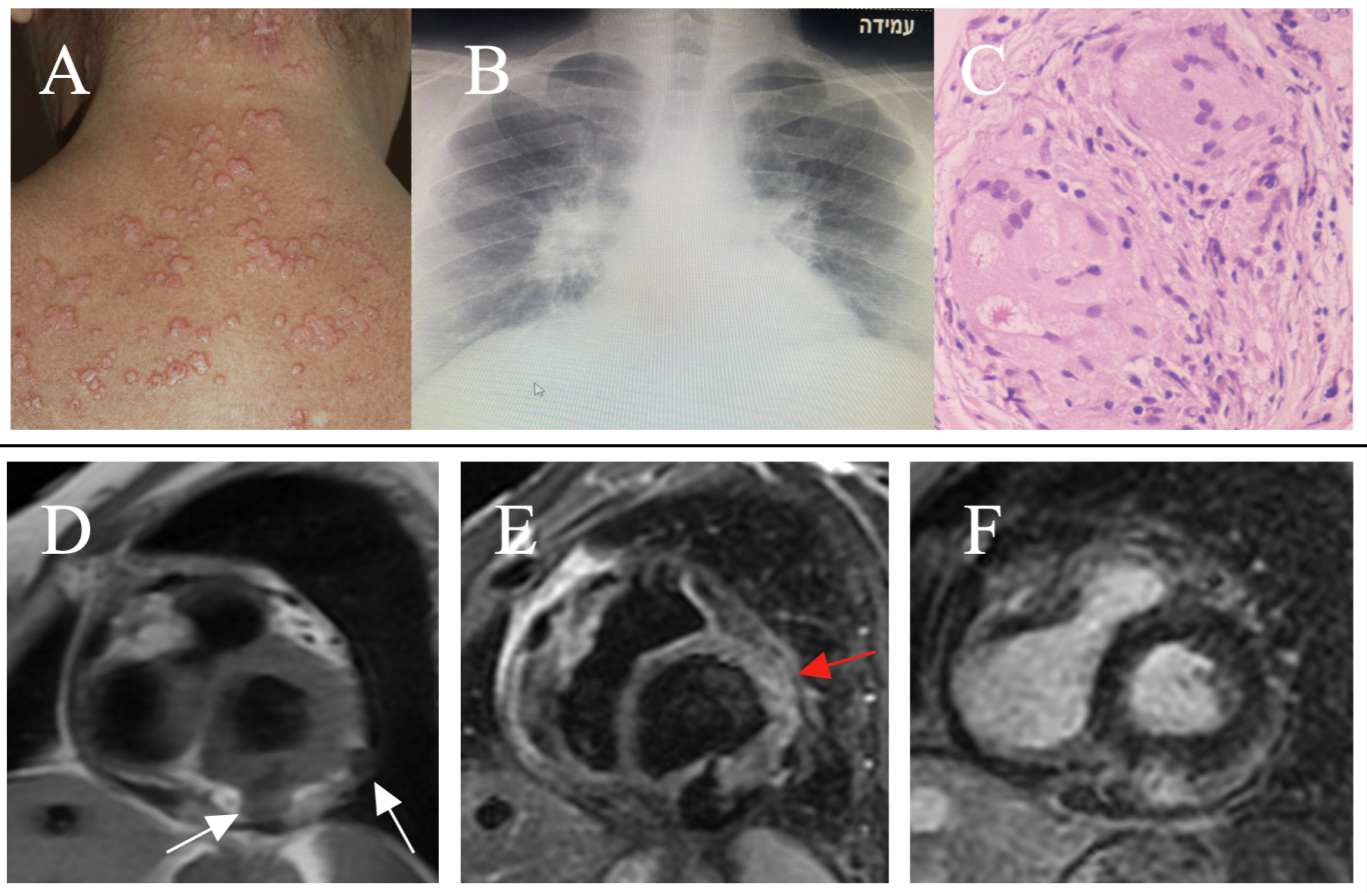
**Clinical-instrumental findings in Cardiac Sarcoidosis**. Multiple and papular skin lesions on the trunk (A – adapted from Torquato MF *et al*. [64]). Bilateral hilar lymphadenopathy at CXR in pulmonary sarcoidosis (B). Hematoxylin and eosin staining of myocardial sample shows non necrotizing granuloma (C - adapted from Markatis E *et al*. [65]). CMR shows in short axis view, two transmural fibrocalcific nodular lesions (white arrows) localized at inferior and lateral basal left ventricular segments. Pathological findings appear isointense in T1-FSE (D), hypointense in T2-STIR (E) and patchy hyperintense in T1-GRE-IR for detection of LGE (F). Moreover, T2-STIR shows in antero-lateral segments, mild hyperintensity due to edema (red arrow). Two stages coexist (chronic and active inflammation). CXR, chest X-ray; CMR, cardiac magnetic resonance; T1-FSE, T1-weighted fast spin echo sequence; T2-STIR, T2-weighted short tau inversion recovery sequence; T1-GRE-IR, T1-weighted gradient echo inversion recovery sequence; LGE, late gadolinium enhancement.

The characteristic chest radiographic feature of pulmonary sarcoidosis is bilateral hilar lymphadenopathy (Fig. 1, Ref. [64, 65]), seen in at least 40% of sarcoidosis patients, frequently accompanied by prominent right paratracheal lymphadenopathy. Enlarged mediastinal, subcarinal, anteroposterior (AP) window, or hilar lymph nodes are observed in approximately 80% of patients with sarcoidosis.

Pulmonary parenchymal infiltrates are found alone in 16% of sarcoidosis cases and coexist with lymphadenopathy in more than 40% of patients. These infiltrates are usually bilateral and primarily located in the central regions and upper lobes of the lungs. Chronic fibrosis can lead to reduced lung volume, retraction of the hilum, distortion of lung architecture, bronchiectasis, formation of bullae, and cystic areas appearing as radiolucencies on imaging [66].

In sarcoidosis, symmetrical lymphadenopathy is a key diagnostic feature; however, asymmetric and bulging lymph node enlargement—typically suggestive of malignancy or tuberculosis—has also been reported [65].

At initial presentation, pulmonary opacities—such as nodules and reticular patterns—primarily affecting the middle and upper lobes, are present in 20–50% of patients. The nodules vary in size and can coalesce and cause alveolar collapse, thus producing consolidation.

CXR has the advantage of being widely available and low-cost, and it exposes patients to a low radiation dose; however, its diagnostic accuracy is approximately 50% [67]. Although the CXR is the standard imaging modality for the initial assessment of pulmonary sarcoidosis, its sensitivity is limited for the detection of small pulmonary nodules, subtle patchy infiltrates, mediastinal lymphadenopathy, and early parenchymal or pleural involvement [68]. CXR is generally useful for detecting early manifestations of the disease, such as hilar lymphadenopathy and pulmonary infiltrates. However, advanced imaging modalities, including high-resolution computed tomography (HRCT), are often necessary to more accurately evaluate the extent of disease involvement, detect subtle parenchymal changes, and distinguish sarcoidosis from other conditions with overlapping radiographic features [69].

According to the 1999 American Thoracic Society (ATS)/European Respiratory Society (ERS)/World Association of Sarcoidosis and Other Granulomatous Disorders (WASOG) guidelines, which remain applicable, there are three main indications for performing a computed tomography (CT) scan in the context of suspected sarcoidosis: (a) when CXR is normal but clinical suspicion persists; (b) in the presence of atypical clinical or radiological findings; and (c) for the evaluation and diagnosis of potential disease-related complications [61].

Despite these guidelines recommendations, recent studies have highlighted the clear advantages of CT over CXR.

HRCT provides superior sensitivity and specificity compared to CXR in detecting thoracic manifestations of sarcoidosis [70]. ^18^F-fluorodeoxyglucose (FDG) PET (positron emission tomography)-CT is valuable in evaluating active inflammation, identifying occult disease sites, and assessing treatment response. However, FDG uptake is not specific to sarcoidosis and may be seen in infections or malignancies [71].

Radiologic findings can mimic other diseases (lymphoma, tuberculosis, hypersensitivity pneumonitis, silicosis, and berilliosis), necessitating careful differential diagnosis [72].

### 5.4 Echocardiographic Imaging

Transthoracic echocardiography is an important noninvasive, low-cost, and readily available modality to guide diagnosis; however, there is no single pathognomonic feature for CS [73, 74].

Despite its low sensitivity and specificity, echocardiography helps rule out other cardiac diseases by excluding valvulopathies, congenital heart diseases, or other forms of cardiomyopathy. For this reason, it could be used as a screening exam in patients with systemic sarcoidosis [47, 75].

Pathological findings, when detectable, reflect infiltration of cardiac structures by granulomatous lesions. The left ventricle (LV) is frequently involved, showing changes ranging from dilation with reduced ejection fraction (EF) to normal findings with preserved systolic function.

Increased cardiac size is a significant determinant of clinical outcomes. In fact, multivariate analysis showed that left ventricular end-diastolic diameter is an independent predictor of all-cause mortality, even after adjusting for left ventricular ejection fraction (LVEF) [76].

Interestingly, when comparing clinical variables between patients with and without pulmonary lesions, those without pulmonary involvement exhibited larger end-diastolic diameters and worse LVEF.

Even when LVEF appears preserved, global longitudinal strain (GLS) can detect subclinical cardiac disease. GLS impairment is independently correlated with late gadolinium enhancement (LGE) burden, all-cause mortality, and pacemaker (PM)/implantable cardioverter defibrillator (ICD) requirement [77, 78].

Wall motion abnormalities vary from diffuse to regional impairment in non-coronary distribution. Aneurysms are more frequently observed in the inferolateral wall, while basal septal thinning or thickening with bright echodensity is suggestive of CS [79, 80].

Like other inflammatory heart diseases, CS can mimic left ventricular hypertrophy (pseudohypertrophy) due to wall edema, although thickening is usually milder compared to eosinophilic or giant cell myocarditis [81, 82].

As with all myocarditis forms, CS may involve not only both ventricles, but also the atria, heart valves, and pericardium.

When the RV shows systolic dysfunction or wall motion abnormalities, it may mimic arrhythmogenic right ventricular cardiomyopathy [83].

However, RV dysfunction less commonly results from direct inflammatory damage than from secondary impairment due to pulmonary hypertension with multifactorial mechanisms [83].

Echocardiographically, pulmonary hypertension may be underestimated due to a poor continuous wave Doppler signal at the tricuspid regurgitation. In such cases, contrast echocardiography with an air-blood-saline mixture can enhance Doppler signal detection [84, 85].

Cardiac valves are not a usual target of sarcoidotic inflammation (<3%), but infiltration of papillary muscles may cause dysfunction of the subvalvular mitral apparatus, leading to secondary regurgitation [31, 86].

Small pericardial effusion is present in about 20% of patients; however, only a few cases with constrictive hemodynamics have been reported [40, 87].

### 5.5 Cardiac Magnetic Resonance Imaging

CMR is a cornerstone for non-invasive evaluation of myocardial inflammation [88].

Thanks to its high spatial resolution, CMR enhances anatomical detail. Moreover, it provides tissue characterization (inflammation and fibrosis), potentially guiding endomyocardial biopsy (EMB) and increasing its diagnostic yield [89].

According to the three consecutive—but sometimes overlapping—histological stages of CS pathology, CMR is useful for identifying early patchy myocardial involvement, including edema, granulomatous infiltration, and fibrosis.

T2-weighted fat-saturation sequences detect myocardial edema in a high percentage (up to 90%) of CS patients. Granulomas may also be visible during the acute phase [90, 91, 92].

Granulomas appear as well-demarcated but uneven lesions, and their characteristics vary depending on the degree of inflammation (Fig. 1).

Another promising tool for identifying inflammatory edema is the T2 mapping sequence, though its diagnostic accuracy still requires further investigation [93].

The immune-mediated damage seen in CS does not follow coronary artery distribution. In one study, Mavrogeni and colleagues [94] identified microvascular heart disease as an early finding in sarcoidosis. They analyzed 43 asymptomatic patients with normal echocardiograms and found that 34 of them had deteriorated myocardial perfusion reserve index and diffuse fibrosis using stress perfusion-fibrosis CMR.

In asymptomatic sarcoidosis patients, myocardial scarring can be detected in 20–25% of cases. Although its clinical significance is uncertain, the prognosis often appears favorable [95, 96].

For this reason, current guidelines do not recommend routine evaluation with CMR or PET/CT in patients without cardiac symptoms and with normal ECG and echocardiogram [97].

Importantly, a proper clinical context is essential to correctly interpret the exam. The diagnostic approach must differ between patients with known CS and those being evaluated for suspected disease.

Athwal and colleagues [98] report that the prevalence of LGE was only 32% among patients undergoing CMR for suspected CS, with the remaining patients showing no signs of tissue damage, even in the small subset (10%) with reduced LVEF.

On the other hand, the presence of LGE negatively affects prognosis. A meta-analysis by Hulten revealed a 29% of patients with LGE experienced a high incidence of cardiac death and VAs [99].

There is no pathognomonic LGE pattern for diagnosing CS. A common finding is a non-ischemic distribution involving the subepicardium and mid-myocardium. However, its absence does not exclude subclinical disease, as demonstrated in biopsy-proven studies [23, 100].

Additionally, a small study revealed a possible basal-to-apex gradient in LGE distribution, with predominant involvement of basal segments [101].

One peculiar, though not pathognomonic, feature is the so-called “hug sign”, which can be detected both on CMR and PET using LGE sequences and ^18^F-FDG uptake, respectively [100]. This sign, observed in the short-axis view, shows enhancement of the basal septal insertion points of both ventricles (anterior and posterior). This typical pattern, although its prevalence is unknown, matches the cardiac scarring described by Tavora and colleagues [102] in a small autopsy study.

Although pericardial involvement in sarcoidosis is typically benign, CMR can assess the pericardium by detecting thickening, inflammation, and effusion [103].

### 5.6 ^18^F-FDG Positron Emission Tomography (PET)

PET imaging has greatly enhanced the identification of myocardial inflammation and the prognostic assessment of CS: an abnormal PET scan is a central criterion in the diagnostic guidelines for CS [7, 104].

PET is a nuclear medicine technique that uses radioactive isotopes combined with a molecule that targets specific tissues to obtain imaging of physiological processes at the molecular level.

The most effective technique for assessing myocardial inflammation is ^18^F-FDG PET; it is a glucose analogue that identifies areas with high glycolytic activity, like inflammatory cells, in particular multinucleated giant cells and lymphocytic infiltration, within granulomas.

However, a key drawback of using ^18^F-FDG as a tracer is its natural uptake by the myocardium, and so ^18^F-FDG PET requires specific preparation before imaging to minimize physiological glucose uptake by healthy heart muscle and highlights myocardial lesions from intact myocardium. Various protocols for preparation and imaging have been applied: the most common strategy is based on optimal dietary preparation with high fat and low carbohydrate diet followed by 12-h fasting before scan [105, 106]; additionally, in certain centers, an intravenous dose of 50 IU/kg heparin is administered before the procedure to acutely elevate free fatty acid levels by activating lipoprotein and hepatic lipase, thereby decreasing glucose uptake by normal heart muscle [43].

In a properly prepared patient, the typical ^18^F-FDG PET image shows no uptake in the myocardium; however, low-level uptake in the lateral wall may be considered normal, especially if it is uniform and not linked to any resting perfusion abnormalities [107]. Diffuse ^18^F-FDG uptake may suggest inadequate suppression of normal myocardial glucose metabolism, or it could reflect the presence of numerous sarcoid granulomas distributed throughout the myocardium. There are no imaging features on ^18^F-FDG PET that are exclusively pathognomonic for CS, and a range of abnormal uptake patterns has been reported: absent uptake, diffuse uptake, focal uptake (“hot spot”), patchy uptake, and focal or diffuse uptake [104].

For accurate diagnosis, an ^18^F-FDG PET study is usually performed in combination with myocardial rest-perfusion imaging with 13N-ammonia or 82-rubidium to locate areas of resting hypoperfusion with non-coronary distribution secondary to microvascular compression from resolved inflammation or scar [100, 108]. Using both techniques (^18^F-FDG PET and perfusion scan), it is possible to discriminate various phases of CS:

• Early phase characterized by isolated active inflammation, showing increased ^18^F-FDG uptake without perfusion defects;

• Progressive inflammation with elevated ^18^F-FDG uptake but no significant perfusion defects;

• Peak active inflammation with high ^18^F-FDG uptake and small perfusion defects;

• Progressive myocardial damage with high ^18^F-FDG uptake and extensive perfusion defects, known as the “mismatch pattern”;

• “Burn-out phase” with fibrotic disease, severe perfusion defects, and minimal or no ^18^F-FDG uptake, indicating non-caseating granulomas retrieval with fibrosis formation [43, 45, 105].

At the time of diagnosing CS, whole-body FDG-PET is the most effective method to exclude extracardiac disease when there is no FDG uptake outside the heart and no signs of skin or eye involvement [109, 110]. Whole-body FDG-PET scans not only detect active inflammation in the heart muscle but also help guide biopsies of extracardiac lesions, with a high success rate of exposing sarcoid histopathology [108, 111]. However, if there is no extracardiac uptake, the specificity of ^18^F-FDG PET for diagnosing CS is reduced [104].

^18^F-FDG PET also plays a role in assessing the prognosis of affected patients: the combination of increased ^18^F-FDG uptake and perfusion defects on PET is linked to a higher risk of death and VT, even after adjusting for LVEF [110]. Moreover, RV inflammatory involvement is shown to have a significant adverse impact on outcomes: these patients experience a five-fold higher event rate compared with those with preserved perfusion and metabolism [112], indicating that focal RV involvement may be a marker of more severe disease. Instead, extra-cardiac ^18^F-FDG uptake is not associated with death or VA [110]. Atrial ^18^F-FDG uptake is predictive of atrial tachyarrhythmias [104, 113].

FDG PET is currently the most reliable non-invasive method for monitoring the disease and guiding immunosuppressive therapy in both CS and extra CS. To assess the response to treatment, follow-up FDG PET scans can be conducted, although the optimal follow-up interval is not firmly established; in practice, these scans are generally repeated every 6–9 months to reassess the level of inflammation [110]. The assessment of response to therapy is analysed visually and quantitatively upon the use of standardized uptake values (with higher sensitivity than qualitative assessment) of ^18^F-FDG; it is calculated as radioactivity concentration in the region of interest relative to the injected dose and body weight. A reduction or an increase in standardized uptake value (SUV) detected on serial PET scans has a prognostic value, although there is no definitive SUV cutoff that can reliably distinguish inflamed myocardial tissue from normal tissue [108]. A significant decrease in SUV in patients treated with steroids indicates a reduction of active inflammation, and so treatment could be tapered [27, 105]. Instead, the lack of reduction of SUV is associated with an almost twentyfold increase in risk of major adverse cardiac events, including death, cardiac transplantation, HF hospitalization, and implantable cardiac device (ICD) [114]: so increased SUV requires intensification of therapy [27, 105].

The primary drawbacks of PET include its inability to provide simultaneous tissue characterization and the substantial rate of false-positive findings caused by incomplete suppression of normal myocardial ^18^F-FDG uptake; inadequate patient preparation can affect approximately 15–25% of cases [106, 115]. Further limitations involve the qualitative nature of PET scan interpretations, which are generally reported as simply positive or negative and do not precisely reflect the severity of the disease [100].

For the future, cardiac PET may incorporate novel radiotracers that appear less influenced by physiological myocardial uptake. For example, activated inflammatory cells overexpress on their surface somatostatin receptors (SSTRs), especially SSTR2A; PET tracers targeting the SSTR have been shown to offer a more specific alternative to FDG for imaging CS, and they could have a potential application for the diagnosis and monitoring of CS [116].

Despite the respective limitations of CMR and PET imaging, simultaneous PET/CMR imaging is now available and offers complementary information on disease pathophysiology in a single co-registered scan. Furthermore, a combined CMR/PET allows evaluation of myocardial abnormalities, according to LGE or mapping, to be compared with regions with enhanced myocardial ^18^F-FDG uptake, resulting in greater both specificity and resolution. The sensitivity of PET alone in detecting CS is 0.85, while CMR alone is 0.82. The combined PET/CMR hybrid imaging demonstrates higher sensitivity at 0.94 [112] and is associated with a lower rate of false-positive findings [117].

Moreover, combined cardiac PET/CMR has significantly lower cumulative radiation dose (and this is an important consideration in younger patients), a shorter imaging acquisition compared with separate sequential CMR, FDG-PET, and Single Photon Emission Computed Tomography (SPECT) perfusion and a better experience for the patients [118]. Using this combined imaging approach, four distinct patterns can be identified:

• CMR negative/PET negative, absence of CS;

• CMR positive/PET positive with non-ischemic LGE pattern and focal or focal-on-diffuse ^18^F-FDG uptake in case of active disease;

• CMR positive/PET negative in case of chronic disease with myocardial scarring (presence of LGE) but with no increases in ^18^F-FDG uptake;

• CMR negative/PET positive, where ^18^F-FDG uptake is focal, focal-on-diffuse or diffuse despite no LGE findings. This pattern might represent early inflammation stage of CS or incomplete physiological suppression of ^18^F-FDG [115].

### 5.7 Endomyocardial Biopsy

Histopathological diagnosis is currently considered the gold standard for the diagnosis of CS.

A definitive diagnosis of CS can be established when an EMB demonstrates non-caseating granulomas in a patient with suspected disease, provided that other granulomatous diseases have been excluded.

Nevertheless, EMB has limited sensitivity, identifying non-caseating granulomas in less than 25% of cases of CS [119, 120].

In a recent study by Mälkönen H *et al*. [121], the sensitivity of EMB was reported to be 38%, increasing to 49% with repeated sampling. Predictors of a positive EMB included the clinical presentation, as well as the activity, extent, and location of myocardial involvement. Notably, the sensitivity of EMB was higher in patients with more extensive myocardial disease [121].

The low sensitivity of EMB is influenced by various factors, including sampling limitations, the heterogeneous myocardial distribution of granulomas, lesion location, and the stage of disease at the time of biopsy. Typically, tissue samples are obtained from the RV septum because it is safely accessible via conventional transvenous cardiac biopsy. However, sarcoid granulomas more frequently involve the left ventricular wall and the basal interventricular septum—regions that are technically challenging to access with standard biopsy techniques. Moreover, histological findings of CS show a temporal evolution, making the diagnosis difficult from biopsy specimens in some stages of the disease, such as the early interstitial phase or the late fibrous phase [122].

Electroanatomic mapping and image-guided endomyocardial biopsy have been shown to enhance the diagnostic yield of EMB in CS [123, 124].

Although the diagnostic yield of EMB in CS is limited, histological differentiation—such as distinguishing CS from giant cell myocarditis—is crucial for guiding therapeutic strategies and determining prognosis.

Therefore, the American Heart Association/American College of Cardiology Foundation/European Society of Cardiology (AHA/ACCF/ESC) scientific statement regarding the role of EMB in the management of cardiovascular diseases considers EMB to be reasonable in cases of suspected CS. Specifically, it is indicated in clinical scenarios involving unexplained HF of at least three months’ duration accompanied by a dilated LV, new VAs, Mobitz type II second- or third-degree AVB, or failure to respond to standard therapy within one to two weeks [125].

## 6. Diagnostic Algorithms

The diagnosis of CS presents a significant clinical challenge because of the absence of uniform guidelines, particularly in patients without extracardiac involvement or with isolated CS.

Over the years, several diagnostic criteria have been proposed: the World Association of Sarcoidosis and Other Granulomatous Disorders (WASOG) was the first to develop a sarcoidosis organ assessment Instrument [126]. Subsequently, the HRS guidelines were published in 2014 [47], and more recently the Japanese guidelines [7] (Table 1).

**Table 1.** **Summary of diagnostic criteria for clinical diagnosis of CS**.

WASOG criteria	Presence of extra-CS and one or more of the following:
	∙ Treatment-responsive cardiomyopathy and AVB
	∙ Reduced LVEF in the absence of other clinical risk factors
	∙ Spontaneous or inducible sustained VT with no other risk factor
	∙ Mobitz type II or 3rd degree heart block
	∙ Patchy uptake on dedicated cardiac PET
	∙ Delayed enhancement on CMR
	∙ Positive gallium uptake
	∙ Defect on perfusion scintigraphy or SPECT scan
	∙ T2 prolongation on CMR
HRS criteria	Histological diagnosis of extra-CS and one or more of the following:
	∙ Corticosteroid- and/or immunosuppressant-responsive cardiomyopathy or heart block
	∙ Unexplained reduced LVEF (<40%)
	∙ Unexplained sustained (spontaneous or induced) VT
	∙ Mobitz type II or 3rd degree heart block
	∙ Patchy uptake on dedicated cardiac PET (in a pattern consistent with CS)
	∙ Late gadolinium enhancement (LGE) on CMR (in a pattern consistent with CS)
	∙ Positive gallium uptake (in a pattern consistent with CS)
JCS criteria	Criteria for cardiac involvement: the presence of cardiac involvement is strongly suggested if two or more of the five major criteria are met, or if at least one major criterion and two or more of the three minor criteria are fulfilled.
	Major criteria
	(a) High-grade AVB (include complete AVB) or fatal VA (e.g., sustained VT, and ventricular fibrillation)
	(b) Basal thinning of the ventricular septum or abnormal wall anatomy (ventricular aneurysm, thinning of the middle or upper ventricular septum, regional ventricular wall thickening)
	(c) LV contractile dysfunction (LVEF <50%) or focal ventricular wall asynergy
	(d) ^67^Ga citrate scintigraphy or ^18^F-FDG PET reveals abnormally high tracer accumulation in the heart
	(e) Gadolinium-enhanced CMR reveals delayed contrast enhancement of the myocardium
	Minor criteria
	(f) Abnormal ECG findings: VAs (nonsustained VT, multifocal or frequent premature ventricular contractions), bundle branch block, axis deviation, or abnormal Q waves
	(g) Perfusion defects on myocardial perfusion scintigraphy (SPECT)
	(h) EMB: monocyte infiltration and moderate or severe interstitial fibrosis

CS, cardiac sarcoidosis; WASOG, World Association of Sarcoidosis and Other Granulomatous Disorders; AVB, atrioventricular block; LVEF, left ventricular ejection fraction; VT, ventricular tachycardia; VA, ventricular arrhythmias; FDG, fluorodeoxyglucose; PET, positron emission tomography; HRS, Heart Rhythm Society; ECG, electrocardiogram; EMB, endomyocardial biopsy; JCS, Japanese Circulation Society; SPECT, Single Photon Emission Computed Tomography.

According to the WASOG criteria, the HRS guidelines, and the Japanese guidelines, there are two pathways for diagnosing CS: histological diagnosis and clinical diagnosis.

The histological diagnosis requires the presence of noncaseating granulomas on histological examination of the myocardium with no alternative cause identified.

For clinical diagnosis, both the WASOG criteria and the HRS guidelines require the presence of granulomatous inflammation in an organ other than the heart, and one or more clinical or imaging criteria [47, 126].

The Japanese guidelines are unique because they do not necessarily require histological confirmation of sarcoidosis for the clinical diagnosis; therefore, the clinical diagnosis group includes patients with negative findings on EMB or patients who did not undergo EMB. Cardiac sarcoidosis may be diagnosed clinically: (1) if epithelioid granulomas are detected in organs other than the heart and clinical features strongly indicate cardiac involvement; or (2) when there are clinical findings strongly indicative of pulmonary or ocular sarcoidosis; at least two of the five characteristic laboratory findings associated with sarcoidosis [(1) Bilateral hilar lymphadenopathy; (2) High serum angiotensin-converting enzyme (ACE) activity or elevated serum lysozyme levels; (3) High serum soluble interleukin-2 receptor (sIL-2R) levels; (4) Significant tracer accumulation in ^67^Ga citrate scintigraphy or ^18^F-FDG PET; (5) A high percentage of lymphocytes with a CD4/CD8 ratio of >3.5 in broncho-alveolar lavage (BAL) fluid]; and there are clinical findings highly suggestive of cardiac involvement [7].

The peculiarity of the Japanese guidelines is that they also include a diagnostic algorithm for isolated CS. Also in this case, a distinction is made between a histological diagnosis group and a clinical diagnosis group (isolated CS is diagnosed clinically when the criterion (d) and at least three other criteria of the major criteria (a) to (e) are satisfied).

In a recent work of Nentwich K *et al*. [127], histological analysis of EMB showed focal infiltration of CD3-positive T lymphocytes in >90% of the patients, even in the absence of non-caseating granulomas. Therefore, inflammation in EMB with elevated CD3-positive T lymphocytes should arouse suspicion for possible diagnosis of CS, and it could be used as a new surrogate histological finding [127].

Thus, rather than thinking about the diagnosis of sarcoidosis in a binary way (positive or negative), it is more useful to think about the probability of CS, where clinical, laboratory, and multimodal imaging findings must be integrated.

## 7. Treatment

### 7.1 Medical Management

The initiation of treatment should be based on the risk-benefit ratio.

In case of symptomatic patient, treatment should be started if the diagnosis of sarcoidosis is definite and highly probable; if the diagnosis is probable, the risks versus benefits of treatment should be thoroughly discussed with the patient; in the possible/low probability group treatment should not be performed due to the uncertainty of the diagnosis and the potential risks of treatment; in case of unlikely CS, there is no indication for immunosuppressive treatment [4].

Although there are no randomized trials on corticosteroids in the CS, they remain the mainstay of therapy, and they are currently considered the first-line treatment.

In patients with clinically relevant CS, evidenced by functional cardiac abnormalities such as heart block, arrhythmias, or cardiomyopathy, the European Respiratory Society (ERS) clinical practice guidelines strongly recommend treatment with corticosteroids, with or without additional immunosuppressive agents, despite the very low quality of supporting evidence [128].

Currently, there are no standardized protocols outlining the initiation, tapering, or maintenance of corticosteroid therapy. The 2016 Japanese Circulation Society Guidelines recommend initiating therapy with prednisone at a dose of 30 mg daily (0.5 mg/kg/day) or 60 mg every other day (1.0 mg/kg every other day) for the first four weeks. Thereafter, the dose should be tapered by 5 mg daily or 10 mg every other day at intervals of 2 to 4 weeks, aiming to maintain a dose of 5–10 mg daily or 10–20 mg every other day [7].

In individuals with life-threatening manifestations or rapidly progressive disease, high-dose intravenous methylprednisolone should be started (500–1000 mg/day IV for 3–5 days followed by oral prednisone) [4].

In patients with frequent symptomatic VA and evidence of myocardial inflammation, immunosuppressive therapy may be administered in combination with antiarrhythmic drugs to reduce the arrhythmic burden [129].

Evidence regarding the impact of corticosteroids on long-term outcomes remains conflicting. In the systematic review of Sadek MM *et al*. [130], which aimed to systematically review the published studies assessing the effects of corticosteroid therapy in patients with CS, 47.4% of patients with AV conduction disease improved after treatment; however, the effect of corticosteroid treatment on left ventricular function was uncertain, as well as on ventricular arrhythmias and mortality [131].

Second-line immunosuppressive agents, which include methotrexate, azathioprine, mycophenolate mofetil, leflunomide, and cyclophosphamide, are initiated when corticosteroids are insufficiently effective or their dose must be reduced to spare the patient from their toxic effects. Patients undergoing nonsteroidal immunosuppressive therapy should be followed up with care because of their potential adverse effects, some of them fatal [132].

The recommended dose of methotrexate is 10 to 20 mg/week, while the recommended dose of azathioprine is 50 to 200 mg daily.

Tumor necrosis factor (TNF) inhibitors have shown potential as therapeutic agents in sarcoidosis, particularly for refractory cases or when the use of corticosteroids and alternative steroid-sparing therapies is limited by contraindications or poor tolerability [133].

The monoclonal anti-TNF antibody infliximab has been shown to be effective in refractory CS, and it is also well tolerated in patients with LV dysfunction [134, 135].

Adalimumab, a fully human anti-TNF agent, can be an alternative administered subcutaneously [136, 137].

Some data indicate that rituximab (anti-B-cell therapy) may be a therapeutic option for refractory CS [138].

### 7.2 Invasive Management

In general, the management of CS involves immunosuppressive therapy to control inflammation, combined with cardiac-specific pharmacological and non-pharmacological treatments, including device implantation and heart transplantation. Patients with CS and ventricular dysfunction are treated according to current guidelines, using the same medications and devices recommended for the general HF population.

VA, the second most common CS manifestation after AV abnormalities, more often follows a macro-reentrant circuit in granulomatous infiltration or scar, but active inflammation might also cause VA by triggered activity and increased automaticity [100]. CS patients usually have multiple VT morphologies (more than with other cardiomyopathies) caused by the patchy nature of sarcoid infiltration and the overlap between inflammation and scarring that occur in the ventricular myocardium. However, common sites of involvement identified are the para-tricuspid area and the RV apex.

Immunosuppressive therapy combined with antiarrhythmic drugs can decrease the burden of VAs in patients with frequent VT and signs of active myocardial inflammation [139]. Catheter ablation, often guided by endocardial mapping, could help control arrhythmias that do not respond to medical therapy; however, its effectiveness in treating VT in CS is variable [140], and CS patients undergoing VT ablation have higher recurrence rates than those with other cardiomyopathies, despite successful ablation: about half of patients must undergo than one ablation procedure [51, 141]. In cases resistant to both medication and ablation, bilateral cardiac sympathectomy may be considered [142].

ICD implantation is a class I indication for all patients who have survived sudden cardiac arrest or experienced sustained VT, as well as for those with an LVEF of 35% or less despite optimal medical therapy [143].

Determining which CS patients are at increased risk of sudden cardiac death is still a significant clinical challenge. Patients with clinically manifest CS have an approximate 10% risk of sudden cardiac death over five years of follow-up, whereas the risk in those with subclinical CS is not well established but is probably much lower [144]. After a CS diagnosis, risk stratification of VA is a necessity; however, the risk of sudden cardiac death is significant (9%) in CS presenting with lone high-grade AVB (also without VT) and normal LVEF [145]. Moreover, whenever there is a need for permanent pacemaker implantation, even if bradyarrhythmia is reversed with immunosuppression, a preventive ICD implantation should be considered, regardless of LVEF [143]. ICD implantation should also be considered for CS patients with LVEF >35% who exhibit significant LGE on CMR after resolution of acute inflammation, and for those with an LVEF of 35–50% and a single morphologic VT inducible on programmed electrical stimulation [143].

In CS patients in the end-stage phase of HF, despite medical and interventional therapies, ventricular assist device and heart transplant should be considered. Patients with CS who undergo heart transplantation achieve acceptable long-term outcomes that are comparable or better than those of other patient populations. Furthermore, there is no evidence of sarcoidosis recurrence in the graft when patients are maintained on low-dose corticosteroids [146, 147, 148].

It remains uncertain whether atrial arrhythmias in CS arise directly from inflammation and/or fibrosis or whether they occur secondary to left ventricular dysfunction. No dedicated guidelines exist for managing atrial arrhythmias in CS, other than the general recommendation to avoid class I antiarrhythmic agents due to their potentially harmful effects in structural heart disease. Nevertheless, catheter ablation may be beneficial both for symptom relief and for preventing inappropriate ICD therapies [27].

## 8. Conclusions

The diagnosis of CS is still challenging, despite the emerging role of new diagnostic techniques that can provide valuable information for diagnostic purposes. Increased awareness of the disease is essential to begin the diagnostic process, which may include ECG, echocardiography, CXR, and chest HRCT as an initial step. In case of high clinical suspicion, second-level imaging techniques can be used to confirm the diagnosis (CMR, ^18^F-FDG PET, and EMB). A timely diagnosis is important to start effective therapies.

Because many gaps in knowledge persist, randomized clinical trials are needed to understand who should be treated, which therapeutic strategy is preferable, and the optimal duration of treatment.
